# Long-Term Outcomes After Epigastric Hernia Repair in Women—A Nationwide Database Study

**DOI:** 10.3389/jaws.2023.11626

**Published:** 2023-09-25

**Authors:** M. W. Christoffersen, N. A. Henriksen

**Affiliations:** ^1^ Digestive Disease Center, Bispebjerg Hospital, University Hospital of Copenhagen, Copenhagen, Denmark; ^2^ Department of Gastrointestinal and Hepatic Diseases, Herlev Hospital, University of Copenhagen, Herlev, Denmark

**Keywords:** primary ventral hernia, recurrence, female patients, mesh repair, sutured repair

## Abstract

**Aim:** Women have the highest prevalence of epigastric hernia repair. Outcomes after epigastric hernia repair are rarely reported independently, although pathology and surgical techniques may be different than for other primary ventral hernias. The aim of this study was to evaluate long-term outcomes after epigastric hernia repairs in women on a nationwide basis.

**Methods:** Nationwide cohort study from the Danish Hernia Database. Complete data from women undergoing elective epigastric hernia repair during a 12 years period (2007–2018) was extracted. A 100% follow-up was obtained by combining data from the National Civil Register. The primary outcome was operation for recurrence, secondary outcomes were readmission and operation for complications. Outcomes for open sutured repair, open mesh repair mesh, and laparoscopic repairs were compared.

**Results:** In total, 3,031 women underwent elective epigastric hernia repair during the study period. Some 1,671 (55.1%) women underwent open sutured repair, 796 (26.3%) underwent open mesh repair, and 564 (18.6%) underwent laparoscopic repair. Follow-up was median 4.8 years. Operation for recurrence was higher after sutured repairs than after open mesh and laparoscopic repairs (7.7% vs. 3.3%, vs. 6.2%, *p* < 0.001). The risk of operation for complications was slightly higher after open mesh repair compared with sutured repair and laparoscopic repair (2.6% vs. 1.2%, vs. 2.0%, *p* = 0.032), with more operations for wound complications in the open mesh group (2.0%, *p* = 0.006).

**Conclusion:** More than half of the women underwent a suture-based repair, although mesh repair reduces risk of recurrence. Open mesh repair had the lowest risk of recurrence, but on the expense of slightly increased risk of wound-related complications.

## Introduction

Women have the highest prevalence of epigastric hernia repair [1, 2] and outcomes after epigastric hernias are not well investigated as an entity [3].

Primary ventral hernias in women may have a different epidemiology due to pregnancy with the rapid increasing pressure on the abdominal wall combined with the hormonal-induced softening of the connective tissue leading to widening of the linea alba [4]. Recent European and American guidelines suggest postponing elective ventral hernia repair in women of childbearing age until after the last pregnancy and then perform a mesh-based hernia repair [5]. In line with this, an epidemiological study found that epigastric hernia repair had the highest prevalence in women of 41–50 years of age [1], where most women presumably have completed planned pregnancies. Although gender disparities in the surgical field has not previously been elucidated recent large-scaled studies have showed that women had a significantly higher risk of recurrence and complications after ventral hernia repair, regardless of the surgical technique and was less likely to receive a mesh-based repair [2, 6].

The aim of this study was to evaluate long-term outcomes after epigastric hernia repairs in women on a nationwide basis.

## Methods

This was a nationwide cohort study based on prospectively registered data from the Danish Hernia Database. The Danish Hernia Database provide detailed intraoperative data such as timing of repair (elective/emergency), primary or recurrent repair, defect size, type of mesh, sutures, and fixation. The inclusion period covered a 12 year period from 1st January 2007 to 31st December 2018. Exclusion criteria were umbilical or incisional hernias, male patients (phenotypic), patients undergoing recurrent repairs, and hernia repairs performed in relation to other surgical procedures. The follow-up period was defined as time from the primary operation until operation for recurrence, death, emigration, or end of study period (31st December 2018). Data from the Danish Hernia Database were supplemented with data from the National Civil Register, ensuring a 100% follow-up on deaths, immigration, mortality, readmittance, reoperation for complications, and reoperation for recurrence.

Moreover, the National Civil Register provides information regarding the patients American Society of Anesthesiologists score (ASA), comorbidities [Charlson Comorbidity Index (CCI)]. Operation for recurrence was defined as a subsequent operation for an epigastric hernia after a previous similar epigastric hernia repair (defined as an operation for a recurrent epigastric hernia in Danish hernia Database). We included only one (the first) operation for recurrence for each patient. Any additional operations for recurrence were not included in the analysis. The hernia size was defined as the widest diameter of the hernia defect measured intra-operatively by the surgeon registered in the Danish hernia Database.

The types of hernia repairs were divided into open or laparoscopic repair, and subgroup analyses were made for the open repairs (mesh vs. sutured repair).

### Statistics

For statistical analysis we used statistical software package IBM statistical software package SPSS version 28. Observation time adjusted estimates of reoperation rates (cumulated reoperation rates) were obtained using the Kaplan-Meier method and presented as a cumulated hazard function and compared with log rank-test. Additionally, subgroup-analysis was made for the EHS size classification for primary ventral hernias, different techniques, mesh positioning (inlay/plug, sublay, onlay and, intraperitoneal), and for the different suture materials (non-absorbable, slowly absorbable, and fast absorbable). The statistical method used (Kaplan-Meier) ensure that the rate of recurrence is relative to the number of patients at risk.

Pearson Chi-Square Tests was used to compare the groups regarding to surgical technique and a multivariate multiple logistic regression or Cox regression analysis was performed for identification of independent covariates. Univariate covariates expressing a *p*-value lesser than 0.2 were entered simultaneously into the multiple logistic regression (or Cox regression model when appropriate). Presented hazard ratios (HR) were adjusted for possible contributions for other variables. A *p*-value < 0.05 was considered significant. Data are presented as median with range and percentages with 95% confidence intervals (95% CI), if not stated otherwise. Data are presented as median (range) and percentages with 95% CI, if not stated otherwise.

## Results

**FIGURE 1:**
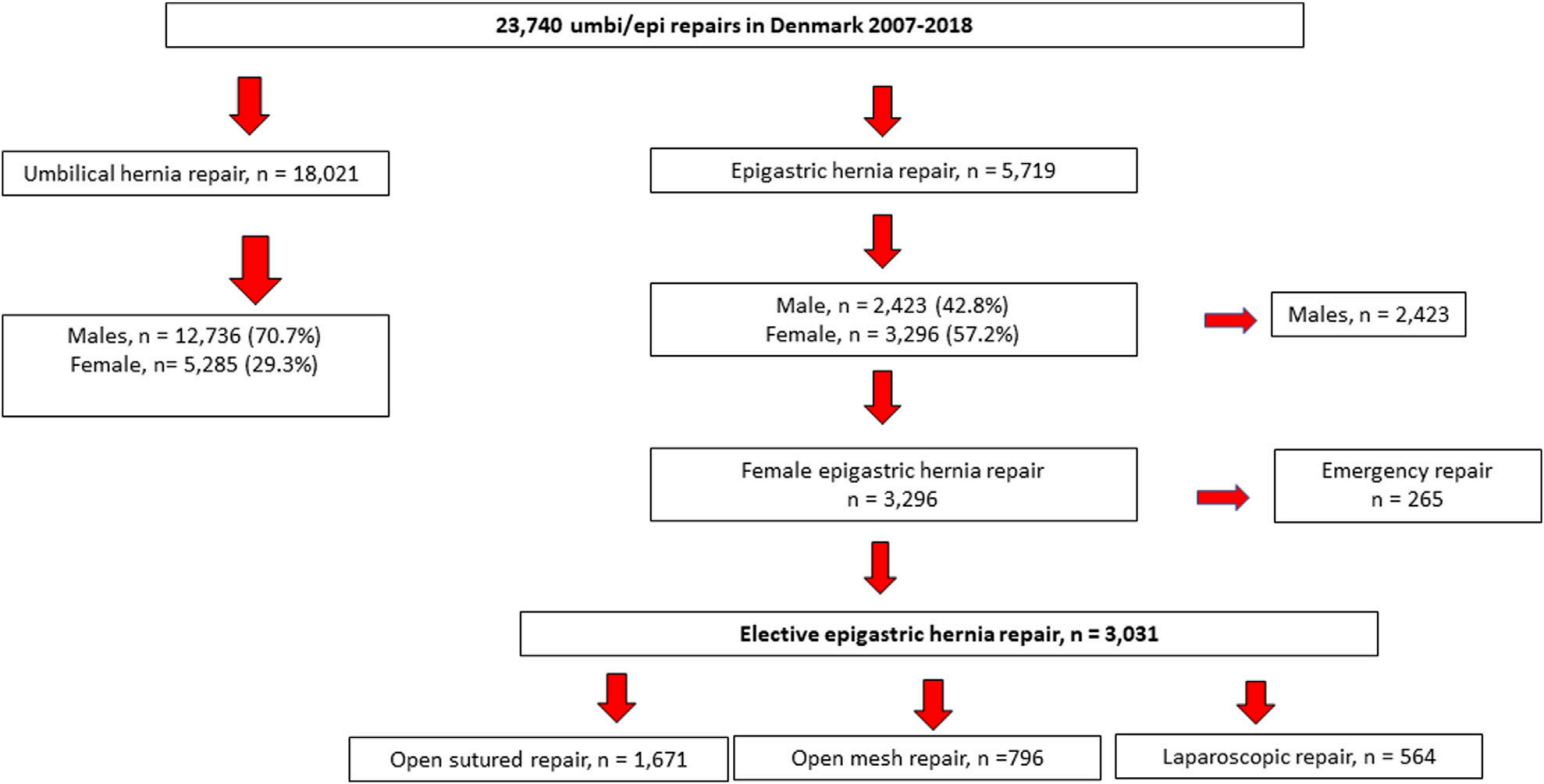
Flowchart depicting the study cohort.

During the study period, a total of 23,740 patients underwent primary ventral hernia repair and the 18,021 patients who underwent umbilical hernia repair were excluded from the analysis. Some 5,719 patients underwent epigastric hernia repair and 57.2% were women and 42.8% were male patients. A total of 3,031 women underwent elective epigastric hernia repair and were included in the analysis (Figure 1). A 100% follow-up on readmission, operations for complications, and recurrence was obtained, and follow-up was median 4.8 years (range: 2.6–7.1 years).

More than half of the women [*n* = 1,671, (55.1%)] underwent a sutured repair. Open mesh repair was performed in 796 (26.3%) of the women, whereas only 564 (18.6%) underwent laparoscopic repair. The mean defect length was 1.81 cm (95% CI = 1.63–1.99) and mean width was 1.72 (95% CI = 1.55–1.90). Further demographic and intraoperative details are depicted in Table 1.

**TABLE 1:** Patient demograpics.

	Open suture, *n* = 1,671, (55.1%) n (%)	Open mesh, *n* = 796, (26.3%) n (%)	Laparoscopic = 564, (18.6%) n (%)
Age (years)			
18–39	586 (35.1)	183 (23.0)	107 (19.0)
40–50	501 (30.0)	204 (25.6)	123 (21.8)
51–60	350 (20.9)	200 (25.1)	164 (29.1)
61–93	234 (14.0)	209 (26.3)	170 (30.1)
Charlson Comorbidity Index			
0	1,414 (84.7)	590 (74.5)	409 (72.5)
1	170 (10.2)	118 (14.9)	94 (16.7)
2	67 (4.0)	45 (5.7)	39 (6.9)
3	18 (1.1)	39 (6.9)	22 (3.9)
Hernia defect size^a^			
0–1 cm	1,419 (84.9)	403 (50.6)	82 (14.5)
>1–4 cm	225 (13.5)	371 (46.6)	411 (72.9)
>4 cm	27 (1.6)	22 (2.8)	71 (12.6)
Defect closure			
No closure	—	340 (42.7)	386 (68.4)
Sutured closure	1,671 (100)	456 (57.3)	178 (31.6)
Suture for defect closure			
Fast absorbable	59 (3.5)	13 (2.8)	3 (1.7)
Slowly absorbable	208 (12.5)	69 (15.0)	28 (15.7)
Non-absorbable	1,397 (84.0)	378 (82.2)	147 (82.6)
Mesh placement			
Onlay	—	299 (37.4)	0 (0)
Intraperitoneal	—	191 (24.1)	533 (94.5)
Preperitoneal	—	143 (17.8)	28 (5.0)
Retromuscular	—	131 (16.5)	3 (2.2)
Other	—	33 (4.1)	0 (0)
Mesh fixation			
No fixation	—	4 (0.5)	4 (0.7)
Glue	—	2 (0.3)	21 (3.7)
Sutures	—	771 (96.9)	12 (2.1)
Clips	—	6 (0.8)	2 (0.4)
Tacks	—	8 (1.0)	516 (91.5)
Other	—	5 (0.6)	9 (1.0)

^a^ European Hernia Society classification.

**FIGURE 2:**
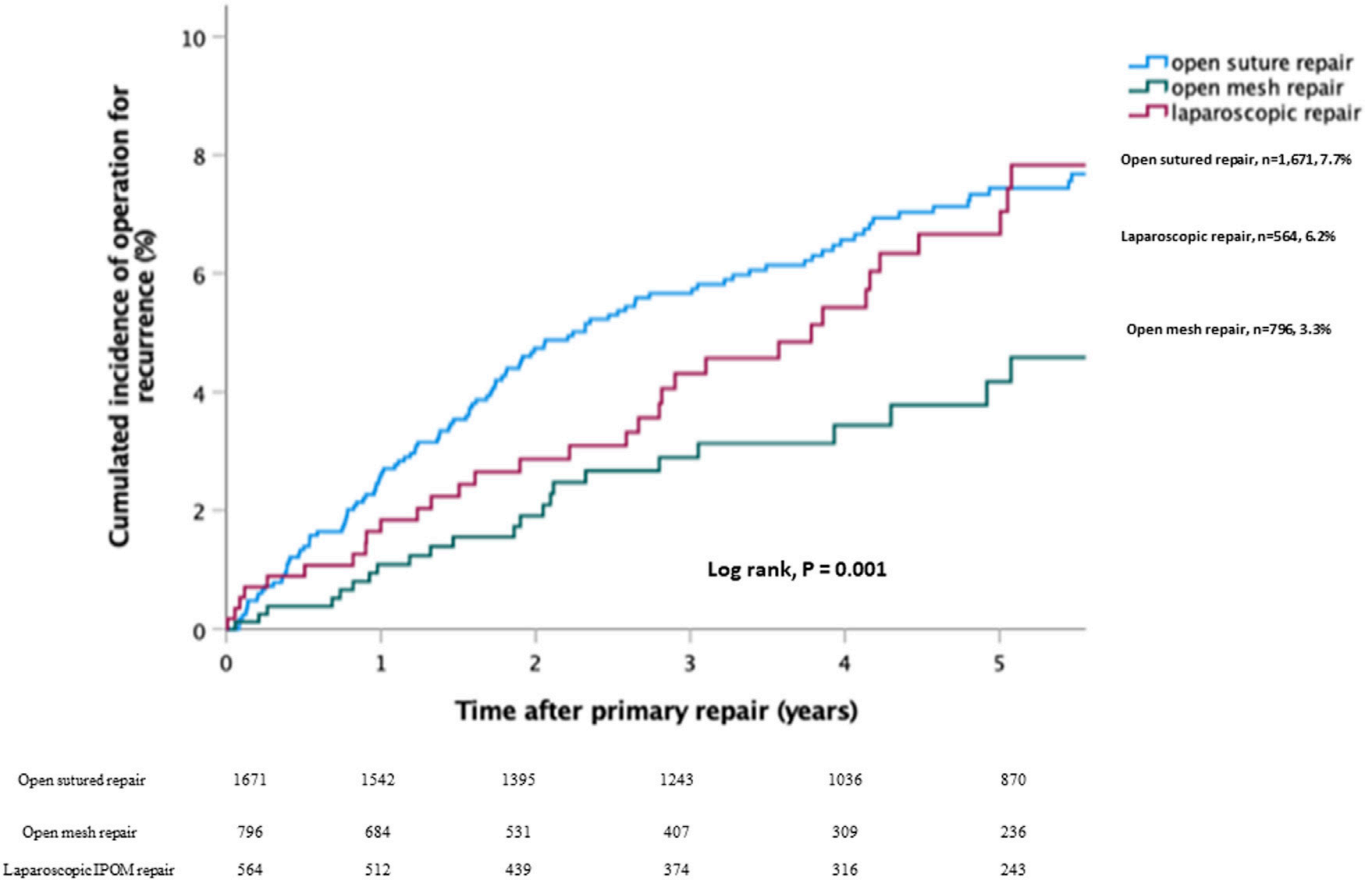
Kaplan-Meier plot illustrating the cumulated risk of recurrences for open sutured repair vs. open mesh repair vs. laparoscopic repair.

The long-term risk of recurrence was lowest after open mesh repair [*n* = 26/796, (3.3%)], laparoscopic repair [*n* = 35/564, 6.2%], and highest after sutured repair (*n* = 129/1,671, 7.7%, *p* < 0.001) (Figure 2). However, operation for complication was slightly but significantly higher after open mesh repair (*n* = 21/796, 2.6%, *p* = 0.032), mainly due to wound complications (2.0% vs. 0.4% and 0.5%, *p* < 0.0001) (Table 2). The readmission rate was highest after laparoscopic repair (62/564, 11.0%, *p* < 0.0001). The most frequent reason for readmission was postoperative pain (23/564, 4.1%, *p* < 0.001).

**TABLE 2:** Readmission and reoperation for complication within 90 days after elective epigastric hernia repair.

	Open sutured repair [*n* = 1,671 (55.1%)] n (%)	Open mesh repair [*n* = 796 (26.3%)] n (%)	Laparoscopic repair [*n* = 564 (18.6%)] n (%)	*p*-value
Readmission	88 (5.3)	61 (7.7)	62 (11.0)	<0.001
Cause of readmission				
Pain	11 (0.7)	9 (1.1)	23 (4.1)	<0.001
Heamatoma/bleeding	7 (0.4)	10 (1.3)	4 (0.7)	
Wound infection	5 (0.3)	10 (1.3)	0 (0)	
Postop care and rehabilitation	12 (0.7)	3 (0.4)	5 (0.9)	
Sepsis	4 (0.2)	0 (0)	1 (0.2)	
Ileus/subileus	0 (0)	0 (0)	1 (0.2)	
Constipation	1 (0.1)	0 (0)	2 (0.4)	
Medical diagnoses^a^	48 (3.0)	29 (3.6)	92 (3.7)	
Operation for complications	20 (1.2)	21 (2.6)	11 (2.0)	0.032
Cause of reoperation				
Deep bleeding	2 (0.1)	2 (0.3)	1 (0.2)
Wound complication	7 (0.4)	16 (2.0)	3 (0.5)	0.006
Laparoscopy	2 (0.1)	1 (0.1)	3 (0.5)	
Bowel resection	1 (0.1)	0 (0.0)	0 (0.0)	
Endoscopic procedure	8 (0.5)	1 (0.1)	3 (0.5)	
Drainage of the abdominal cavity	0 (0.0)	1 (0.1)	1 (0.2)	

^a^ Pneumonia, urinary tract infection, cardiac complications, hepato-biliary, electrolyte derangement, diarrhea, dermatological disease, neurological diseases.

In the multivariable analysis, significant risk factors for recurrence were sutured repair and reoperation for complications within 90 days (OR: 2.2, CI: 1.4–3.6, *p* = 0.03) (Table 3).

**TABLE 3:** Uni- and multivariable analysis. Risk factors for recurrence.

	Univariate analysis HR (95% CI)	*P*	Multivariate analysis HR (95% CI)	*P*
Age, quartiles, years				
18–39	1	0.004	1	
40–50	0.59 (0.40–0.85)	0.005	0.647 (0.40–0.84)	0.272
50–60	0.66 (0.45–0.96)	0.028	0.499 (0.44–0.95)	0.236
61–93	0.53 (0.34–0.82)	0.004	0.579 (0.32–0.79)	0.202
Charlson Comorbidity Index				
0 = no	1	0.319	1	
1 = mild	1.39 (0.94–2.06)	0.098	1.601 (1.07–2.39)	0.126
2 = moderate	0.96 (0.47–1.96)	0.912	1.136 (0.55–2.33)	0.050
3 = severe	1.49 (0.66–3.38)	0.337	2.071 (0.89–4.79)	0.026
Defect size				
0–1 cm	1	0.325		
1–4 cm	1.25 (0.93–1.69)	0.135		
>4 cm	1.13 (0.55–2.32)	0.736		
Use of mesh	0.68 (0.50–0.92)	0.013	0.690 (0.50–0.94)	0.021
Sutured repair				
Open vs. Lap repair	1.02 (0.71–1.46)	0.91		
Readmission within 90 days	1.23 (0.73–2.08)	0.444		
Reoperation for complications within 90 days	2.78 (1.55–4.99)	<0.001	2.91 (1.61–5.24)	0.026

## Discussion

This nationwide study of more than 3,000 women undergoing elective epigastric hernia repair revealed that less than half of the women underwent a mesh-based repair. Open mesh repair had the lowest risk of recurrence, but on the expense of a slightly increased risk of operation for wound-related complications. Readmission was significantly higher after laparoscopic repair compared with both open techniques, mainly due to postoperative pain. Surprisingly, were recurrence rates after laparoscopic repair higher than after open mesh repair—but this result may be biased by defect size and/or body mass index in the laparoscopic group, since more women in the laparoscopic group had defects >4 cm (Table 1).

Several previous studies found a benefit of mesh reinforcement in even the smallest primary ventral hernias [7, 8]. Accordingly, the high rate of sutured repairs in the present study is perturbing. A recent Swedish, nationwide cohort study found that women undergoing umbilical hernia repair had higher risk of recurrence [2]. In relation to this, an American retrospective quality database study analyzing outcomes from >5,000 patients demonstrated that women were less likely to have a mesh-based repair and that women had higher risk of adverse events [6]. A recent propensity-score matched study from the German Herniamed registry found that female patients had higher risk of chronic pain after elective epigastric hernia repair, but with no other differences in outcomes [9]. Other previous studies have shown that rates of complications, hospital readmission, and poor quality of life are higher among females following ventral and incisional hernia repair [10–12]. These findings should encourage future studies on causes and solutions to sex disparities in hernia repair.

Although, causes often are multifactorial, one explanation for the high rate of sutured repair could be the fact that pregnancy increases the risk of recurrence and thus, it is suggested to postpone ventral hernia repair until after the last pregnancy [13]. However, a Danish epidemiological study showed that epigastric hernia repair in women was performed most frequently at the age of 41–50 years, where women most likely are post pregnancies where mesh repair should be the preferred choice [5]. The findings of the present, and other studies may reflect a reluctance to use mesh in female patients, even after pregnancies. Whether this is due to a fear of mesh-related complications, or a presumption that suture is enough, by either patient or surgeon, can only be speculated. Whether these differences are a result of sex disparities in patient -and hernia-related risk factors, or different choices of techniques are not clear, but pose an interesting topic to highlight in future studies.

The higher risk of recurrence after sutured repair compared with mesh repair may be on the expense of a slightly higher risk of wound complications. These findings could argue that a sutured repair could be first choice of repair in patients with low risk of recurrence in shared decision making, as well as patients with high risk of recurrence should be advised repair with mesh, and patients with risk factors for wound complications should be offered minimally invasive repair. Accordingly, surgical societies recommend using a mesh-based repair to reduce recurrence rate, and to choose a minimally invasive approach to decrease the risk of surgical site infection. The present findings of a higher risk of readmission due to postoperative pain after laparoscopic repair, may have driven the shift in many surgical societies from IPOM repair to other new minimally invasive approaches [14–16]. There are several concerns when choosing the optimal repair technique, and both patient-and hernia-related factors, as well as the local expertise may influence outcomes.

This study is strengthened by a large sample size based on nationwide real-life data. However, there are limitations to database studies. First, recurrences in this study were registered as reoperation for recurrence, which highly underestimated clinical recurrence [8, 17]. Second, there were no data on smoking status and body mass index, as these variables were not registered in the Danish Hernia Database until 2018, which could potentially could have impacted the results regarding complications and recurrence. Furthermore, are the reasons for choosing specific procedure and the use of mesh or not, are not registered in the database. Given the nature of a database cohort study the data reflects real-world data and thus, may be biased by surgeons’ preferences regarding defect size and type of repair.

Future large-scaled studies investigating different patient categories with different risk patterns are warranted.

## Conclusion

Epigastric hernias are more frequently performed in women. Nationwide data found that less than half of the women underwent a mesh-based repair, although mesh repair significantly lowered recurrence rate. However, open mesh repair also slightly increased risk of operation for wound complications.

## Data Availability

The raw data supporting the conclusion of this article will be made available by the authors, without undue reservation.
